# Structure-Based Inhibitor Discovery of Class I Histone Deacetylases (HDACs)

**DOI:** 10.3390/ijms21228828

**Published:** 2020-11-22

**Authors:** Yuxiang Luo, Huilin Li

**Affiliations:** 1 School of Pharmaceutical Sciences, Sun Yat-sen University, No.132 Wai Huan Dong lu, Guangzhou Higher Education Mega Center, Guangzhou 510006, Guangdong, China; luoyx69@mail2.sysu.edu.cn; 2 Guangdong Key Laboratory of Chiral Molecule and Drug Discovery, School of Pharmaceutical Sciences, Sun Yat-sen University, Guangzhou 510006, Guangdong, China

**Keywords:** Class I histone deacetylases, structural studies, selective inhibitors, HDAC complexes, drug mechanism

## Abstract

Class I histone deacetylases (HDACs) are promising targets for epigenetic therapies for a range of diseases such as cancers, inflammations, infections and neurological diseases. Although six HDAC inhibitors are now licensed for clinical treatments, they are all pan-inhibitors with little or no HDAC isoform selectivity, exhibiting undesirable side effects. A major issue with the currently available HDAC inhibitors is that they have limited specificity and target multiple deacetylases. Except for HDAC8, Class I HDACs (1, 2 and 3) are recruited to large multiprotein complexes to function. Therefore, there are rising needs to develop new, hopefully, therapeutically efficacious HDAC inhibitors with isoform or complex selectivity. Here, upon the introduction of the structures of Class I HDACs and their complexes, we provide an up-to-date overview of the structure-based discovery of Class I HDAC inhibitors, including pan-, isoform-selective and complex-specific inhibitors, aiming to provide an insight into the discovery of additional HDAC inhibitors with greater selectivity, specificity and therapeutic utility.

## 1. Introduction

Histone deacetylases (HDACs) are enzymes involved in epigenetic regulation through controlling the acetylation state of lysine side-chains in histone tails [1], leading to chromatin condensation and gene transcription repression [2,3]. Additionally, HDACs can indirectly regulate other post-translational modifications (PTMs) through releasing acetyl group from lysine so that other PTMs, for instance, ubiquitination, can mark on the loci [1]. So far, 18 human HDACs have been identified according to their sequence homologies to yeast and are divided into four classes: Class I (HDAC1-3 and 8), Class II with two subclasses (Class IIa includes HDAC4, 5, 7 and 9 and Class IIb corresponds to HDAC6 and 10) and Class IV (HDAC11) are zinc-dependent enzymes (also referred to as classical HDACs family), while Class III are NAD^+^-dependent which called sirtuins (SIRT1-7) [4,5]. Each class has different biological functions [6]. Classes I, II and IV are metal-dependent HDACs that use a metal−water as the nucleophile during catalysis, which is activated via a general acid−base mechanism [7].

Famous as a cancer target, abnormal function and expression of HDACs have been observed in various tumor cells, including breast, lung, liver and gastric, where they are aberrantly recruited to gene promoters [8,9,10]. Except for this, HDACs play roles in neurodegenerative diseases such as Alzheimer’s disease, Huntington’s disease, Parkinson’s disease and mood disorders [11], as well as in HIV infection [12], kidney diseases [13] and inflammatory diseases [14].

Luckily, these abnormalities can be altered by HDAC inhibitors (HDACis). First discovered as inducers of cell growth processes, HDACis show great potential in inhibiting HDACs activity and treating many diseases [6]. Some of them have been approved by the U.S. FDA (Food and Drug Administration), although they are pan-inhibitors with little or no specific isoform selectivity [15]. Here, we specifically focus on the most promising drug targets, Class I HDACs. Except for HDAC8 that is fully active in isolation, HDAC1, 2 and 3 form the catalytic subunit of multiprotein complexes to mediate gene transcription [16]. More specifically, HDAC1 and HDAC2 form the catalytic core of multiple corepressor complexes, including NuRD (nucleosome remodeling and deacetylase), Sin3 (switch intensive 3) and CoREST (corepressor of RE1-silencing transcription), MiDAC (mitotic deacetylase), while HDAC3 forms the key component of SMRT/NCoR (silencing mediator of retinoic acid and thyroid hormone receptors/nuclear receptor corepressor) [15]. As part of these complexes, the HDACs become maximally activated and are targeted to specific regions of chromatin.

In this review, after briefly introducing the enzyme mechanism of Class I HDACs, we specifically focus on the structures of Class I HDACs and their complexes and summarize the development of some representative pan-, isoform-selective and complex-selective inhibitors and their mechanism insights. We aim to provide an up-to-date reference for targeted design and screening of Class I HDACis.

## 2. HDACs

### 2.1. HDACs Substrates

HDACs are capable of catalyzing the removal of the *N*-acetyl group from acetylated lysine residues in histones and non-histone proteins [17,18]. The substrates of HDACs are rather complicated, owing to the overlapping functions of different HDACs and different substrate preferences within HDAC complexes [19]. When one HDAC is knocked down, its activity can be replaced by other isoforms [1]. However, for HDAC8, there is no evidence for histones being its substrates in vivo [17]. Additionally, HDACs impact the functions of more than 50 non-histone substrates (e.g., p53, NF-κB, STAT3 and Hsp90) that regulate cellular development, proliferation, differentiation and death [20]. For example, the acetylation/deacetylation of tumor suppressor protein p53 regulates its transcriptional activity and is related to apoptosis and autophagy, which plays critical roles in eliminating tumor cells [21]. Inhibiting HDACs allows p53-induced transcription kept in an active state, leading to tumor cell death [2,21]. Another example is the signal transducers and activators of transcription 3 (STAT3), which is found highly expressed in diffuse large B-cell lymphoma (DLBCL), regulates gene expression with the aid of HDACs [22]. As HDACs involve different types of substrates, the abnormalities of them correlate with diseases to varying degrees [1,8].

### 2.2. FDA Approved HDAC Inhibitors

HDACis can antagonize the function of HDACs, increase the level of acetylated histones, and show potential towards cancers, neurological diseases, inflammatory diseases, and so on [23,24,25]. In tumor cells, HDACis induce cell apoptosis, cell cycle arrest, senescence, differentiation, autophagy and increase tumor immunogenicity [8]. For example, inhibitors such as SAHA and sodium butyrate (NaB) inhibit cell proliferation, arrest cell cycle at G0/G1 phase, and induce mitochondrial related apoptosis in triple-negative breast cancer (TNBC) cells [26]. Additionally, HDACis also affect the immune system and tumor microenvironment by regulating the differentiation, function and survival of different immune cells, thus inhibiting tumor angiogenesis and metastasis/invasion [1,8]. In neurological diseases, HDACis can induce neuroprotection and the expression of neurotrophins [8,27].

**Figure 1:**
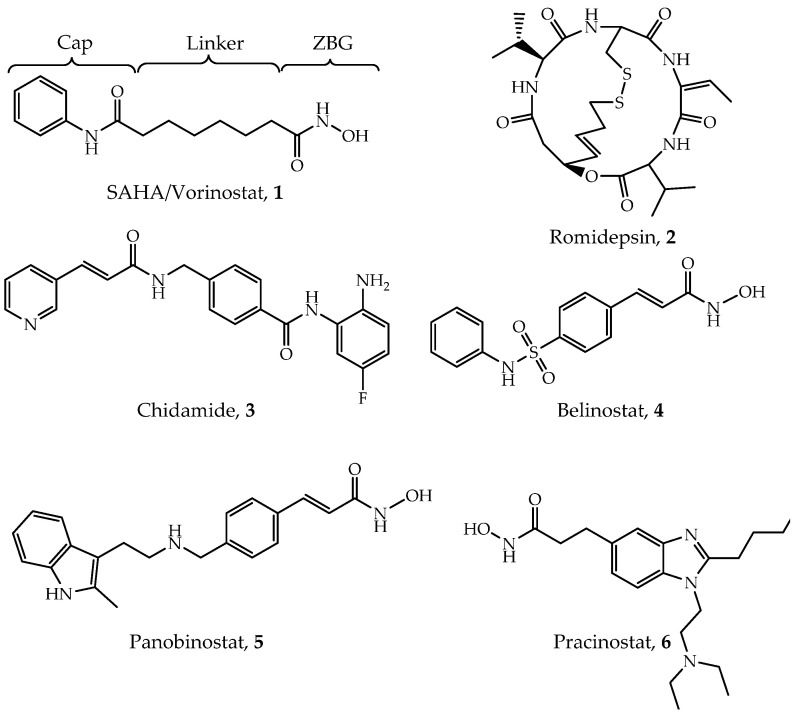
Structures of Food and Drug Administration (FDA)-approved Class I histone deacetylases (HDAC) inhibitors.

Through the unremitting efforts of investigators over the years, six HDACis have been approved by the FDA mainly for cancer treatment (Figure 1), and many others are in clinical trials [28]. The first drug, vorinostat (suberoylanilide hydroxamic acid, SAHA, Compound **1**), was approved by the FDA for the treatment of refractory cutaneous T-cell lymphoma (CTCL) in 2006 [28,29]. A series of clinical trials have confirmed its effects and toxicity in the treatment of CTCL [30,31,32]. In addition, SAHA has shown effectiveness in a variety of solid and hematological tumors such as head and neck cancer [33], Hodgkin lymphoma (HL) and DLBCL [34]. The second drug, romidepsin (FK228, Compound **2**), was reported in 1994 and approved in 2009 [29,35]. Based on two large phase II studies [36,37], romidepsin has been used in the treatment of relapsed or refractory peripheral T-cell lymphomas (PTCL) [38,39]. Chidamide (Compound **3**) was approved by the Chinese National Medical Products Administration (NMPA) for the treatment of relapsed or refractory PTCL in 2014 [40,41] and breast cancer (combined with exemestane) in 2019 [42]. Belinostat (Compound **4**) is also used for treating relapsed or refractory PTCL [43,44]. A phase II study led to FDA approval of belinostat [45]. Panobinostat (Compound **5**) was approved for treating multiple myeloma based on a phase III study (PANORAMA1) [46], while it also shows anti-HIV latency effect in vivo in a clinical trial [47,48]. Pracinostat (Compound **6**) is the latest approved drug for the treatment of acute myeloid leukemia (AML) in 2016 [28,49,50]. Although these inhibitors show quite a promising efficiency in clinical treatment, they exhibit poor selectivity and significant side effects [51,52,53]. Given that, several points pushed the investigation of selective HDACs inhibitors. First, selective HDACs inhibitors may be helpful to reduce those side effects [54]. Second, some isoforms of HDACs are directly related to different types of diseases; thus, treatment may be more specific and effective. For example, PCI-34051 specifically inhibits HDAC8 and has been found to be a specific cytotoxic agent for Jurkat, HuT78 and Molt-4 cell lines [55]. Additionally, it has been reported that selectively inhibiting HDAC3 may help to discover some antiatherosclerotic drugs [28,56]. Therefore, there is an urgent need to develop isoform-selective or complex-specific HDAC inhibitors.

## 3. Structures of Class I HDACs

### 3.1. Structure and Catalytic Mechanism of Monomeric HDACs

**Figure 2:**
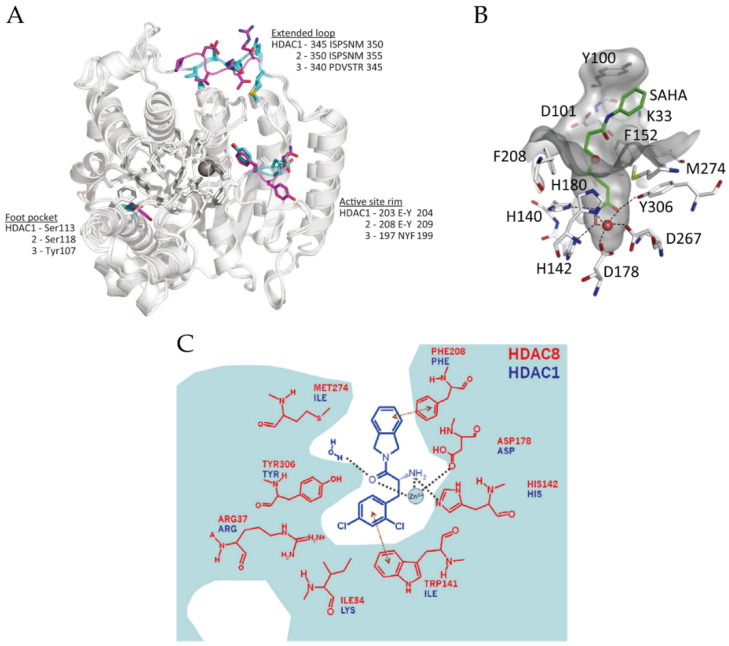
(**A**) Superposition of the structures of HDAC1–3 (PDB (Protein Data Bank) codes: 5ICN, 4LY1 and 4A69). Significant residue differences are highlighted in cyan (HDAC1 and 2) and magenta (HDAC3). Adapted with permission from Millard 2017 [15]. (**B**) The active site of HDAC8 (PDB code: 4QA2). Adapted with permission from Chakrabarti 2015 [17]. (**C**) The foot pocket of HDAC8. Prominent amino-acid side-chain differences between HDAC8 and HDAC1 in the foot pocket are shown. Adapted with permission from Whitehead 2011 [64].

Structural studies have been particularly useful in understanding and refining the mode of inhibitor binding to Class I HDACs; conversely, inhibitor studies have also promoted our understanding of the structure of HDACs. Since Somoza et al. reported the first crystal structure of HDACs in 2004, to date, most crystal structural information of monomer HDACs is about HDAC8 [57,58,59,60,61]. The global structures of Class I HDACs look similar (Figure 2A) because they all contain a large catalytic domain, which consist of a central parallel β-sheet surrounded by several α-helices linked with loops. In addition, they share a 35–160 amino acid unstructured C-terminal tail except for HDAC8, which is used to recruit protein complexes and to be post-translationally modified [15,57,62]. The active sites of Class I HDACs are almost identical, and the entrance of the active sites located at the surface of these enzymes [15]. In the case of HDAC8, the active site contains an approximate 12-Å-deep narrow hydrophobic tunnel formed by hydrophobic residues Phe152, Phe208, His180, Gly151, Met274 and Tyr306, where a zinc ion lies at its bottom as a member of the catalytic pocket (Figure 2B) [57,62]. The zinc ion is pentacoordinate and bound to Asp178 (Oδ1), His180 (Nδ1) and Asp267 (Oδ1), while the other two coordination sites are occupied by the acetyl moiety (carbonyl oxygen) of the substrate and a water molecule [57].

However, several differences also exist between HDAC8 and HDAC1-3. For example, Loop1 of HDAC8 is shorter than the corresponding one in HDAC1-3 [28]. Additionally, lacking the unstructured C-terminal tail may explain why HDAC8 can work as a monomeric protein [57]. The surface around the active site also plays an important role in substrate-binding [15]. A unique solvent-exposure residue Try198 in the surface of HDAC3, which is near to the active site, may be related to substrate specificity (Figure 2A) [28]. Another structural difference between HDAC3 and HDAC1/2 is the extended loop, which also shows a sequence distinction (Figure 2A) [15]. Moreover, a 14-Å “foot pocket” was found lying perpendicular to the end of the hydrophobic tunnel in Class I HDACs, which may be an exit of the acetate product [63,64]. However, the foot pocket in HDAC8 is narrower than in HDAC1-3 because the large side chain of Trp141 in HDAC8 occupies this space (Figure 2C) [15,64]. Additionally, Ser113/Ser118 of HDAC1/2 is altered to tyrosine in HDAC3, which leads to a steric hindrance so that bulky functional-groups of inhibitors are inaccessible to the foot pocket (Figure 2A) [28].

**Figure 3:**
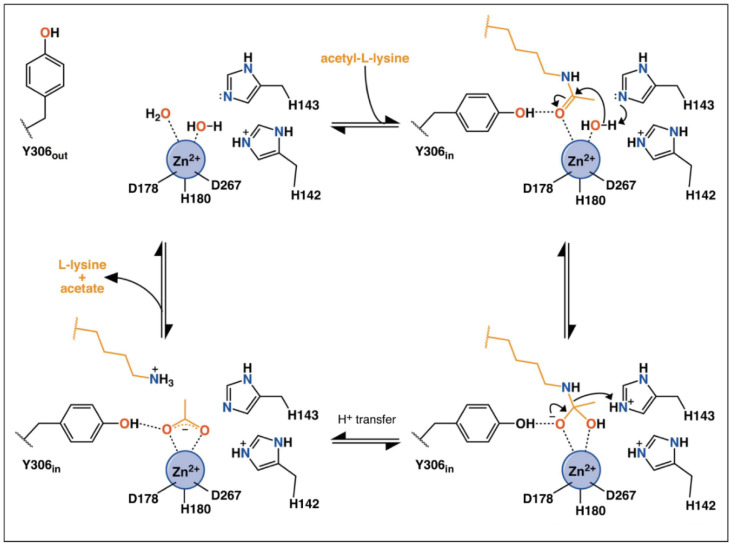
Possible mechanism of catalysis by HDAC8. Adapted with permission from Porter 2019 [66].

Classes I HDACs are metal-dependent enzymes that use a metal−water as the nucleophile during catalysis. The catalytic mechanism of HDACs has been previously summarized by Porter et al. [65,66]. As shown in Figure 3, in a substrate-free state, two of the coordination sites are occupied by water molecules [66]. When the substrate approaches the catalytic pocket, His143 in HDAC8 helps to activate the water molecule, which then nucleophilically attacks the carbonyl carbon. His143 then protonates the amine group and promotes the leaving of deacetylated substrates and acetate. During the catalytic process, Try306 undergoes an induced-fit alteration between “in” and “out” conformations. Meanwhile, His142 stays protonated and maintains the electrostatic environment.

The activity of HDACs can also be regulated by monovalent metal cations or phosphorylation [7,67]. A second metal-binding site was found approximately 7 Å away from the zinc ion in Class I HDACs, which impacts the catalytic mechanism [58,62]. The second metal can be potassium, calcium or sodium ions, depending on the salt contained during crystallization [58]. In the case of HDAC8, when a potassium ion occupies this site, it will be hexa-coordinated by six oxygen from Asp176 (main-chain carbonyl oxygen and Oδ1), Asp178 (main-chain carbonyl oxygen), His180 (main-chain carbonyl oxygen), Ser199 (Oγ) and Leu200 (main-chain carbonyl oxygen), and two of these residues also chelate with zinc ion [62]. A series of computational simulation studies have shown that the second metal-binding site also influences the catalytic pocket through altering the structure of the catalytic site, facilitating the stabilization of deprotonation states of inhibitors [60,68].

Additionally, a unique phosphorylation on Ser39 in HDAC8 impacts protein structure and decreases the enzyme activity [69]. Leng et al. illustrated that phosphorylation of Ser39 distorts the Loop1, which lines at the side of the active site, thus perturbing local structure [7]. It is worth mentioning that there is also phosphorylation at other sites of HDAC1-3 but activating the enzymes, which will be discussed below.

### 3.2. Structure of HDAC Complexes

To date, HDAC8 is the only reported HDACs that can function as a monomer, while all other Class I HDACs must function as a component of multiprotein complexes. The major challenge with structural studies of HDAC complexes is that HDAC1, 2 and 3 work as subunits of large protein complexes and have distinct functions, and can exist in different complexes [15,70]. NuRD, Sin3 and CoREST are the major Class I HDAC multiprotein complexes. Early studies have shown that HDAC1, HDAC2, RBBP4 (RbAp48) and RBBP7 (RbAp46) form the core histone deacetylase complex, which exists in both NuRD and Sin3 macromolecular complexes [71].

**Figure 4:**
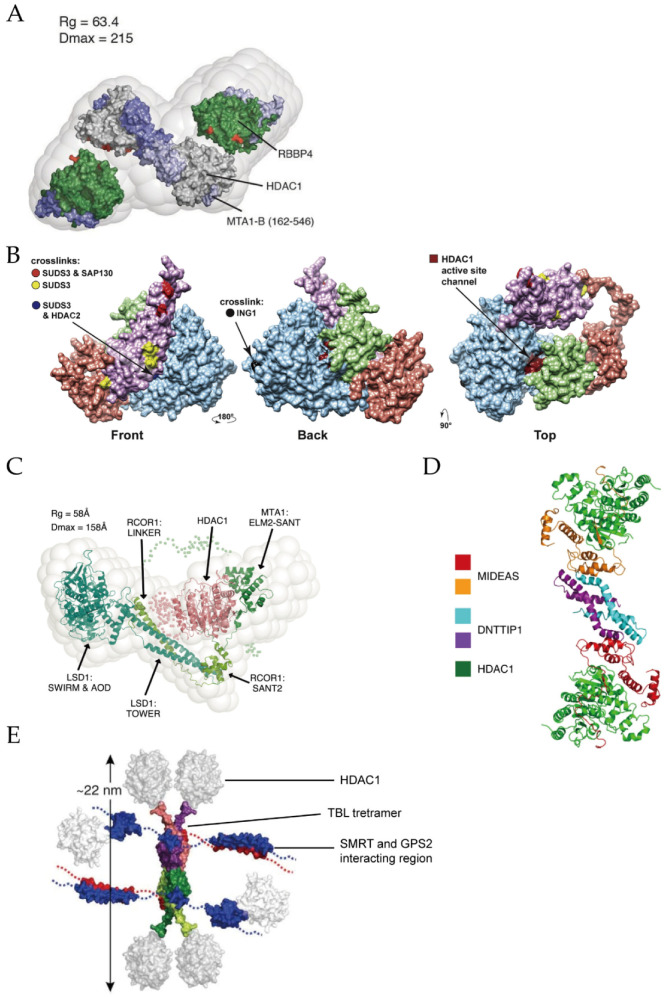
(**A**) Structure of the core NuRD complex (HDAC1/RBBP4/MTA1 (162-546)). Adapted with permission from Millard 2016 [75]. (**B**) Substructure of the Sin3 complex. Adapted with permission from Banks 2020 [80]. (**C**) Structure of the CoREST complex. Adapted with permission from Song 2020 [87]. (**D**) Structure of the MiDAC complex in dimeric form. Adapted with permission from Turnbull 2020 [89]. (**E**) Structure of the SMRT/NCoR complex. Adapted with permission from Oberoi 2011 [93].

The NuRD complex possesses both ATPase and histone deacetylase activities [72], participating in transcriptional repression, chromatin assembly, cell cycle progression and genomic stability [73]. Thus far, at least seven protein families have been found as the components of NuRD: two catalytic subunits including HDAC1/2 and CHD3/4 (chromodomain helicase DNA-binding protein 3/4, also known as Mi-2α/Mi-2β), MTA1/2/3 (metastasis tumor-associated protein 1/2/3), MBD2/3 (methylated CpG-binding domain protein 2/3), RBBP4/7 (retinoblastoma-binding protein 4/7, also called RbAp48/46), GATAD2A/2B (GATA zinc finger domain containing 2A/2B, i.e., p66α/p66β) and CDK2AP1 (cyclin-dependent kinase 2-associated protein 1) [74]. A single-particle negative-stain electron microscopy (EM) method coupled with small-angle X-ray scattering (SAXS) and chemical crosslinking has been applied to reveal the core structure of the NuRD complex [75]. As shown in Figure 4A, its substructure is composed of HDAC1/2, MTA1/2/3 and RBBP4/7 with a binding stoichiometry of 2:2:4. Dimeric MTA1 functions as a backbone to recruit two HDAC1 and four RBBP4 separately and forms an elongated zig-zag conformation through the highly conserved ELM2-SANT and R1/2 domains, of MTA1. Additionally, GATAD2A/B bridges CHD3/4 and MBD2/3, the latter may play a key role in linking them with the core NuRD complex [76]. The detail roles of other subunits in the formation of NuRD complex can be found in a recent review [76].

The Sin3 complex regulates gene transcription at the promoter and transcribed regions, engaging in cell processes such as Notch signaling and mitochondrial functions [77]. Sin3 complex contains Sin3A/B proteins, HDAC1/2, RBBP4/7, SUDS3 (suppressor of defective silencing 3) and SAP30 (sin3-associated protein p30). The Sin3 complex can also be divided into Sin3A or Sin3B complexes depending on which subunit (Sin3A or Sin3B) it contains [78]. Using an affinity purification mass spectrometry (AP-MS) based approach, Washburn’s group revealed that SUDS3 presents in both Sin3A and Sin3B complexes, while SAP30 is only utilized in the Sin3A complex [79]. Later, by integrating chemical crosslinking MS (XL-MS) with AP-MS, they modeled the substructure of the Sin3A complex [80]. Figure 4B shows that Sin3A protein exists as a backbone so that the other subunits, including HDAC1/2, SAP30 and SUDS3, can assemble. The active site of HDAC1 locates at the binding interface of HDAC1 and SAP30. It is important to mention that, when targeting gene, Sin3 requires the aid of additional DNA-binding proteins due to its lack of DNA-binding activity [81].

The CoREST complex has both demethylation and deacetylation activities, silencing the expression of cancer and neurological disorders related genes [82,83]. It consists of CoREST1-3 (also called RCOR1-3) proteins, LSD1 (lysine-specific demethylase 1) and HDAC1/2 [84]. The crystal structure of the LSD1/CoREST revealed the interaction between LSD1 and CoREST, both of them bind to DNA, while the latter also interacts with histones [85,86]. EM study established a bi-lobed structure with LSD1 and HDAC1 at two opposite sides of the CoREST, where RCOR1 acts as a long string linking the other two components (Figure 4C) [87].

The MiDAC complex contains HDAC1/2, DNTTIP1 (deoxynucleotidyl-transferase terminal-interacting protein 1) and the mitotic deacetylase-associated SANT domain (MIDEAS) corepressor protein [15], playing regulatory roles in gene expression of neuronal and embryonic development [88,89]. A previous study suggested that MiDAC is a tetrameric complex that contains four copies of HDAC1/2, DNTTIP1 and MIDEAS, respectively [90]. Cryo-EM structure showed that in the dimeric subcomplex, two HDAC1 are on both sides of MiDAC (Figure 4D) [89]. In addition, the ELM2 domain of MIDEAS does not directly form dimerization as MTA1 in the NuRD complex does. It is DNTTIP1 that actually mediates the dimeric assembly.

The SMRT/NCoR complex is associated with development and homeostasis in inflammation, neuronal and cardiovascular diseases [91]. A highly conserved *N*-terminal region of SMRT/NCoR protein recruits at least three proteins: HDAC3, GPS2 (G-protein pathway suppressor 2) and TBL1 (transducin beta-like 1) [92]. Crystal and NMR structures showed that SMRT interacts with HDAC3 at regions near the active site and the *N*-terminus of TBL1 protein forms a tetrameric interaction with SMRT and GPS2 (Figure 4E) [93,94].

### 3.3. Allosteric Sites and Regulations

**Figure 5:**
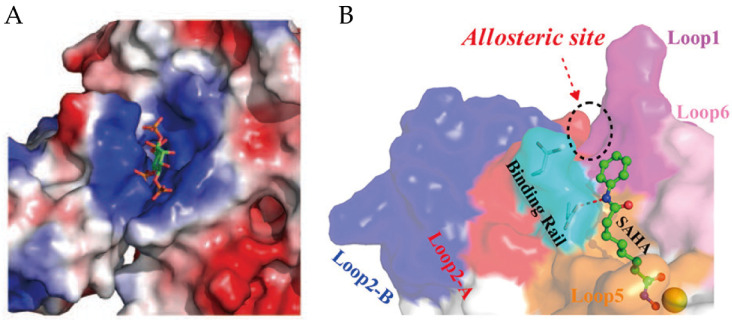
(**A**) The binding site of inositol phosphate with HDAC3-SMRT complex. The inositol phosphate is shown as a stick model. Adaptedwith permission from Watson 2012 [94]. (**B**) Relative position of the allosteric site and binding rail on HDAC2. Adapted with permission from Zhou 2017 [96].

Phosphorylation of HDAC1-3 stimulates the enzyme activity, which is opposite to that of HDAC8 [6]. In addition, the enzymatic activity of HDAC1-3 in complexes has been shown to be regulated by inositol phosphates, which bind in a pocket sandwiched between the HDAC and corepressor proteins [16]. More specifically, in the HDAC3/SMRT crystal structure, inositol phosphate binds to a few conserved key residues (His17, Gly21, Lys25, Arg265, Arg301 in HDAC3 and Lys449, Tyr470, Tyr471, Lys474, Lys 475 in SMRT, Figure 5A) [94,95]. Allosteric communication between the inositol-binding site and the active site has been observed, which facilitates the activation of enzyme activity [16].

Moreover, another allosteric site on the surface of HDAC2 and near the active site of the enzyme (Figure 5B) has been disclosed by computational methods [96,97]. The QM/MM study revealed the flexibility of Loop2 in HDAC2, the conformational changes of X-D dyad in Loop2 (also called binding rail) directly induce the switch of the substrate-binding tunnel [96].

Recently, an NMR study revealed that Helix1-Loop1-Helix2 is an allosteric site of HDAC8 [98]. A bidirectional regulatory effect exists between the Helix1-Loop1-Helix2 region and the active site of HDAC8; thus, Ser39 and Met40 may be key residues in this allosteric regulation. Overall, phosphate-binding sites, binding rail and Helix1-Loop1-Helix2 region may be targets for the design of allosteric inhibitors.

## 4. Pan-Inhibitors

Although high-resolution structures of these Class I HDACs have been determined, developing truly isoform-selective HDAC inhibitors has proven challenging due to the structural similarity of the active sites of these enzymes. Up to date, all six HDAC inhibitors approved by FDA or NMPA are pan-inhibitors [99]. Additionally, a number of pan-inhibitors, such as entinostat (MS-275), are under clinical trials or investigation [100]. A general mechanism of pan-inhibitors to act against Zn^2+^-dependent HDACs is to occupy the active site, thus competitively inhibiting substrate-binding to HDACs [101]. They mainly follow a classic pharmacophore model (Figure 1), consisting of a cap, a linker, and a zinc-binding group (ZBG), which corresponds to surface recognition region, substrate-binding tunnel, zinc chelation site and foot pocket in the target protein, respectively [96]. According to their chemical structures, it can be categorized into four types, including hydroxamic acids, benzamides, cyclic peptides or depsipeptides and aliphatic carboxylic acids [102,103]. Here, we will briefly outline the four types of pan-inhibitors. More pan-inhibitor development can be found in several recent reviews [5,28,99,104].

### 4.1. Hydroxamic Acids

Hydroxamic acids are the most common pan-inhibitors in investigations, represented by SAHA, belinostat, pracinostat, panobinostat, etc. As shown in Figure 2B, the carbonyl oxygen and hydroxyl oxygen of hydroxamic bicoordinates with a zinc ion. Meanwhile, His142, His143 and Try306 (in HDAC8) stabilize the interaction [62,68]. Due to its fair performance in vitro stability, good solubility and easy synthesis, hydroxamic acids are often preferred in the design of novel HDACis [105]. Improved inhibiting effects and selectivity of this category of inhibitors have been demonstrated by modifying the cap and linker region [106]. However, its over-high metal-binding ability can lead to undesirable coordination to other zinc-dependent enzymes such as aminopeptidases, matrix metalloproteinases and carbonic anhydrases, thus causing low selectivity, off-target effects and severe toxicity [99,102,105].

### 4.2. Benzamides

Compared with hydroxamic acids, benzamides such as chidamide and entinostat show better selectivity toward Class I HDACs [107]. In the crystal structure of HDAC2-inhibitor, the primary amine nitrogen and amide oxygen bi-coordinate with zinc ion, but with lower affinity than that of hydroxamic [108]. Meanwhile, the residues surrounding ZBG and foot region form a hydrogen bond network to stabilize inhibitor-binding. Benzamide ring also provides a modifiable site target foot pockets in Class I HDACs [108]. However, its slower binding rate constants may attribute to compromised drug effects.

### 4.3. Cyclic Peptides

Romidepsin is the only approved cyclic peptide inhibitor that is selectivity to Class I HDACs [109]. The mechanism of action of this kind of inhibitors is initiated by the reduction of the disulfide bond, thus releases a thiol group to coordinate the zinc ion in the active site [99,110]. It has been demonstrated that modification on the cap region can increase their biological activity and selectivity [111]. Yet, the toxicity and easy oxidation remain the major challenges in the development of cyclic peptide inhibitors [51,110].

### 4.4. Aliphatic Carboxylic Acids

Low toxicity and easy synthesis are the main features of aliphatic carboxylic acids [19,106]. The specific mechanism of them is not yet clear, most probably through binding to the active site [112]. A docking study also inferred that they might occupy the substrate-binding tunnel as general pan-inhibitors do [113]. Inhibiting HDACs by valproic acid showed potential effects in solid or CNS (central nervous system) tumors and neurological diseases [114,115]. Sodium phenylbutyrate is currently under clinical trials in lymphoma or solid tumors [116]. However, weak zinc-binding ability leads to low inhibitory effects; thus, aliphatic carboxylic acids are often used in combination with other drugs in clinical trials [99].

## 5. Isoform-Selective Inhibitors

A problem with the currently available pan-HDAC inhibitors is that they have limited specificity and target multiple deacetylases, greatly limiting their clinical uses due to significant side effects [117]. The design of isoform-selective inhibitors has become the main focus and is being actively undertaken [112,117,118]. Here, we introduce the current status of selective inhibitor development according to their targeted isoforms.

### 5.1. HDAC8-Selective Inhibitors

HDAC8 is a unique member of Class I HDACs due to its structural distinctions from HDAC1-3. First, as mentioned above, the absence of 35–160 amino acids in the C-terminus may explain why it works as a monomer [17]. Second, the Loop1 of HDAC8 is highly flexible and forms a large part of the active site, which extends to the protein surface; thus, HDAC8 has a wider active site pocket with a larger surface opening than HDAC1-3 [57]. Third, the phosphorylation is also distinctive in HDAC8 than the other isoforms. Moreover, as to the development of HDAC8-selective inhibitors, the exploration of structural features of HDAC8 goes deeper.

**Figure 6:**
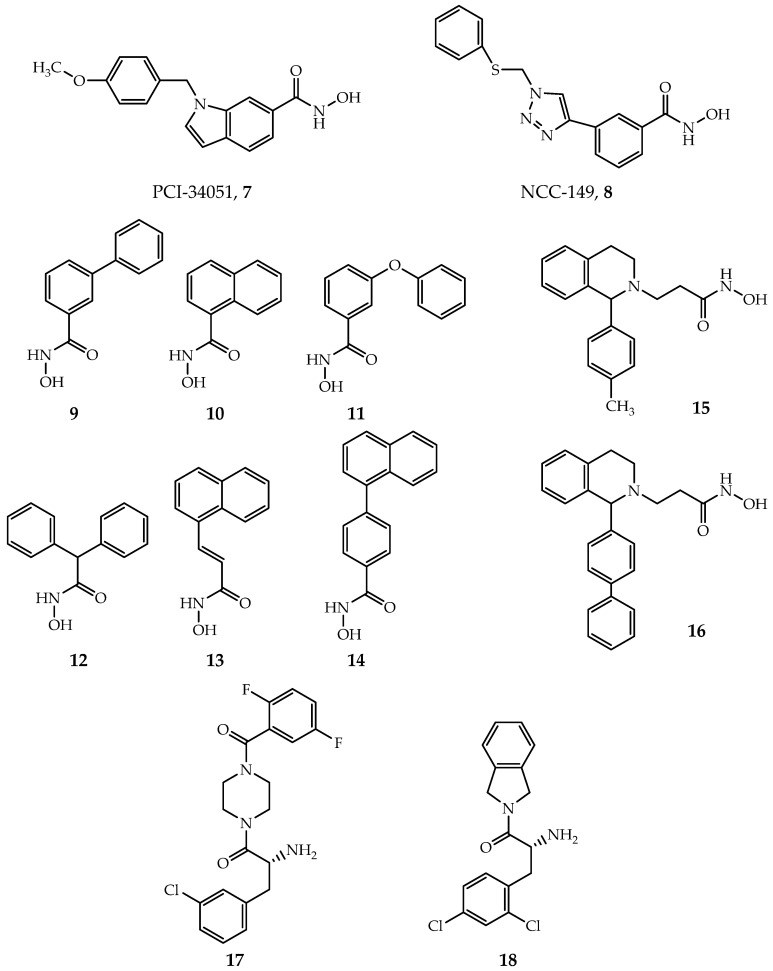
Selective inhibitors of HDAC8.

PCI-34051(Figure 6, Compound **7**) and NCC-149 (Figure 6, Compound **8**) are the most widely investigated HDAC8-selective inhibitors. PCI-34051 has >200-fold selectivity towards HDAC8 over other isoforms; it is found effective or cytotoxic in T-cell lymphoma, leukemia and other types of tumor cells, such as Jurkat, HuT78 and Molt-4 cell [29]. NCC-149 shows a potent inhibiting effect in T-cell lymphoma growth and >500 fold selectivity over HDAC1 and 2, while >30 fold selectivity over HDAC6 in Class IIb [119]. However, the specific mechanism and binding structure of these two inhibitors with human HDAC8 (hHDAC8) are poorly understood. Marek et al. revealed that the binding mode of PCI-34051 and NCC-149 with *Schistosoma mansoni* HDAC8 (smHDAC8) through X-ray crystallization [120]. The two inhibitors coordinate with zinc ion in the active site of HDAC8 by hydroxamic groups, as general hydroxamic acid inhibitors do, but with an “L” shape conformation. The cap group of PCI-34051 and NCC-149 interacts with the Y341 (Y306 in hHDACs) and inserts into the cavity formed by Loop1 and Loop6. All together, they form an HDAC8 selective pocket. In contrast, this pocket is blocked by protruding residues of Loop1 and Loop6 in HDAC1-3. The derivatives of PCI-34051 and NCC-149 are under development, exhibiting great potential [121,122,123].

Most HDACis have a linker group composed of a long fatty chain, as shown in SAHA (Figure 1, Compound **1**). However, Krennhrubec et al. demonstrated that “linkerless” hydroxamic acids (Figure 6, Compounds **9**–**14**) can specifically target HDAC8 [124]. The idea is originated from the discovery of an HDAC8 selective sub-pocket near the active site [57], confirmed by crystal structures [125]. The hydroxamic group binds to zinc ion: meanwhile, the steric hindrance effect of the bulky aryl group of inhibitors causes the split of F152 and M274, exposing the sub-pocket [125]. It is worth mentioning that “linkerless” does not mean that there is no linker region but a much shorter linker. “Linkerless” inhibitors show selectivity towards HDAC8 over HDAC1 and HDAC6 and give prospect in AML, neurodegenerative diseases as well as genetic disorders [124,126].

Additionally, Taha et al. developed a series of HDAC8 inhibitors by modifying the cap region, yielding good selectivity towards HDAC8 over HDAC1-3 (Figure 6, Compounds **15**–**16**) [127]. Their inhibitory effect in neuroblastoma was verified by cellular experiments [127]. By targeting the foot region of HDAC8, Whitehead et al. designed several amino-acid derivatives with HDAC8 selectivity (Figure 6, Compounds **17**–**18**) [64]. Crystal structures revealed that the amide group of inhibitors coordinates with zinc ion, and the foot groups insert into the foot pocket in the active site of protein [64]. The foot pocket in HDAC1 is narrower than in HDAC8, the differences in residues of foot pocket between HDAC8 and HDAC1 may be responsible for the observed isoform selectivity [64]. Furthermore, other HDAC8-selective inhibitors that contain novel active or selective groups are under development [128,129,130,131].

### 5.2. HDAC1/2-Selective Inhibitors

Compared to HDAC8-selective inhibitors, the development of HDAC1/2/3-selective inhibitors has lagged behind owing to their high sequence similarities (85% homological identity between HDAC1 and HDAC2 and 64% between HDAC1 and HDAC3) [6,132]. In addition, fewer structural studies make it even more challenging to design HDAC1/2-selective inhibitors.

**Figure 7:**
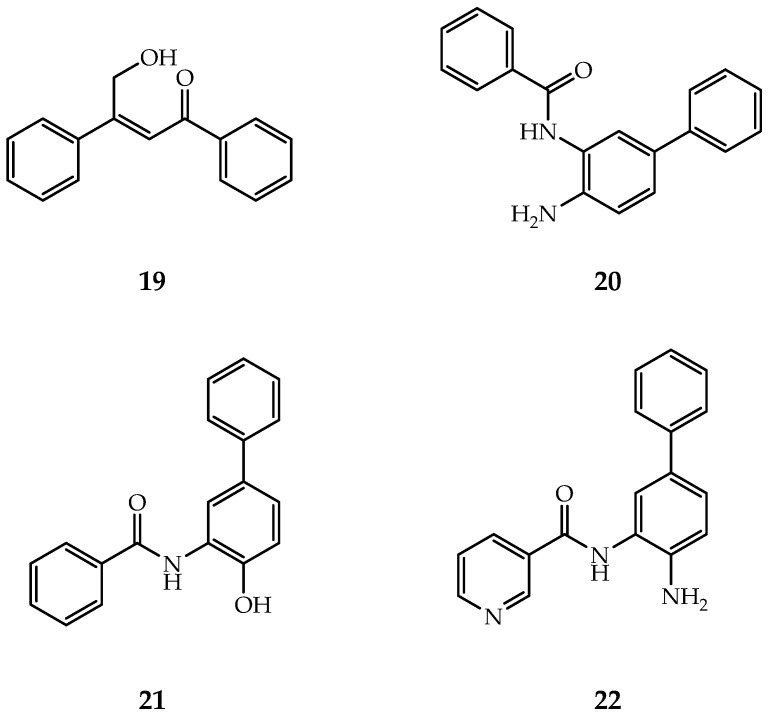
Selective inhibitors of HDAC1/2.

The first HDAC2-selective inhibitor (Figure 7, Compound **19**) was discovered by Zhou et al., whose selectivity depends on inhibition time [133]. The IC50 values show that after 24h inhibition, this inhibitor displays good selectivity towards HDAC2 over HDAC1&3. QM/MM simulation inferred that different ions in the second metal-binding site have different binding kinetics, which leads to the time-dependence selectivity effect of β-hydroxymethyl chalcone inhibitors.

By targeting the foot pocket, Bressi et al. designed a series of *N*-(2-amino-5-substituted phenyl)benzamides (Figure 7, Compound **20**) that are effective to HDAC2, but their selectivity was not fully explored [134]. The crystal structure revealed that the HDAC2 foot pocket consists of Tyr29, Met35, Phe114 and Leu144, and the phenyl groups of inhibitors can insert into this foot pocket [134].

SHI-1:2 is another type of HDAC1/HDAC2-selective inhibitors (Figure 7, Compound **21**–**22**) [132]. The docking study showed that the carbonyl and aniline groups are bound to zinc ion while the phenyl interacts with the foot pocket. However, Try96 in HDAC3 (contains a larger moiety than the corresponding residue, Ser113 in HDAC1) causes this site inaccessible for SHI-1:2.

Additionally, in the substrate-binding tunnel, replacing Glu98 and Try204 in HDAC1/2 with other amino acids may lead to structural distinctions, forming a target for selective inhibitor design [135].

### 5.3. HDAC3-Selective Inhibitors

A structural alignment reveals that five key residues in HDAC3 are different from HDAC1 and HDAC2, including Val13, Leu29, Asp92, Tyr107 and Phe199, which may lead to structural divergence [136]. For example, Try107 in HDAC3 (serine in HDAC1 and HDAC2) causes steric hindrance and thus excludes the binding of inhibitors with large functional groups [136].

**Figure 8:**
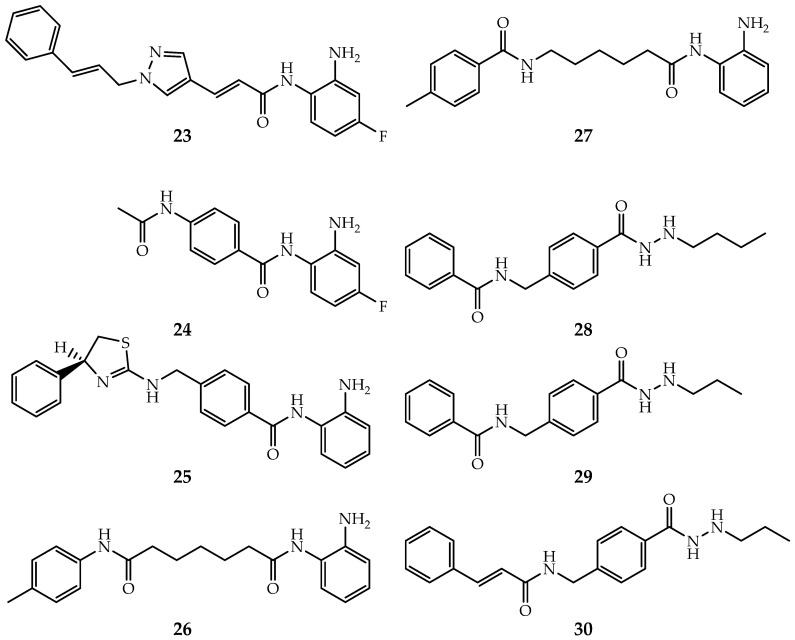
Selective inhibitors of HDAC3.

Benzamide inhibitors show great selectivity in inhibiting HDAC3, thus have earned the favors of investigators [28]. RGFP966 (Figure 8, Compound **23**) is a famous selective inhibitor towards HDAC3 over other Class I HDACs, with effects in hepatoma carcinoma cell [137,138]. BRD3308 (Figure 8, Compound **24**) was first discovered effective in diabetes with >10 folds selectivity over HDAC1 and HDAC2 [139]. A docking study indicated that the selectivity originates from the conformational differences of Try107 and Leu144 [139]. Moreover, Marson et al. discovered an inhibitor (Figure 8, Compound **25**) contained heterocyclic capping group effective for HDAC3-NCoR1 over other monomer Class I HDACs [140]. Moreover, PD-106 (Figure 8, Compound **26**), RGFP109 (Figure 8, Compound **27**), and other benzamides also exhibit HDAC3 selective [141,142,143,144,145]. However, the lack of structural details makes it difficult to gain deep insight into their selective mechanisms.

In addition, McClure et al. developed a group of allosteric inhibitors that show potential in AML (Figure 8, Compounds **28**–**30**) [97]. As suggested by Zhou’s study, these inhibitors may bind to the allosteric site (see Section 3.3) of Class I HDACs, but lead to a close conformation of Phe144 and Phe200 in the substrate tunnel of HDAC3 [96,97]. The specific mechanism of allosteric regulation and inhibition has not yet been disclosed.

## 6. Complex-Specific Inhibitors

Although several HDAC isoform-selective inhibitors have been reported, developing truly isoform-selective HDAC inhibitors has proven challenging due to the structural similarity of the active sites of these enzymes. Given the fact that most of the Class I HDACs must function as a catalytic subunit of gene-regulatory complexes, developing novel inhibitors targeting specific HDAC complexes offers an alternative but yet attractive strategy [15]. Several strategies have emerged for the development of this type of inhibitors, including utilizing specific inhibitor-binding kinetics, developing dual action inhibitors, disrupting protein–protein interactions, and targeting other subunits in HDAC complexes [15,83,146,147,148,149,150,151,152,153,154,155].

**Figure 9:**
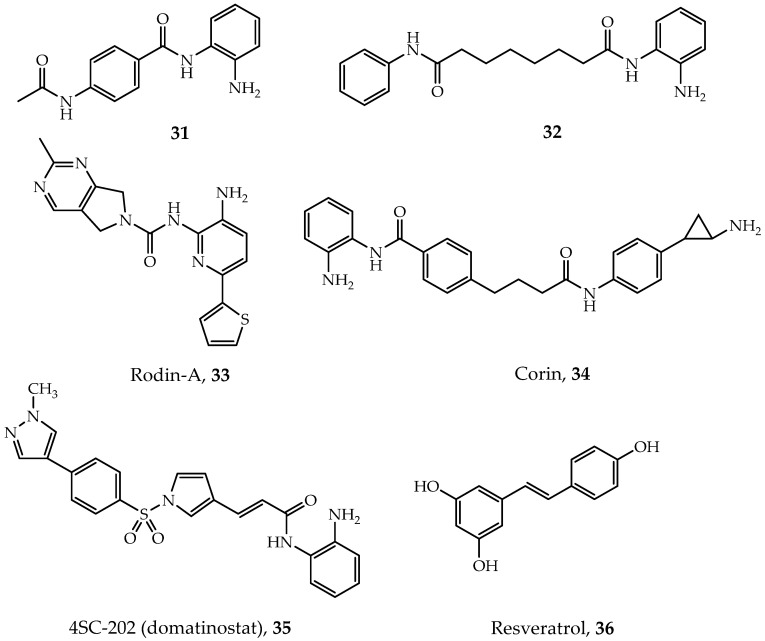
Specific-complex inhibitors.

Using a chemoproteomics method combined affinity capture and quantitative mass spectrometry, Bantscheff and coworkers demonstrated that benzamides inhibitors can have different affinities to distinct HDAC complexes [147,148]. They found that benzamide inhibitors such as CI-994 (tacedinaline, Figure 9, Compound **31**) and BML210 (Figure 9, Compound **32**) are able to bind to the NuRD, CoREST and MiDAC complexes with distinct binding kinetics but exhibit no binding affinity to the Sin3 complex. Fuller et al. discovered a CoREST-specific inhibitor called Rodin-A (Figure 9, Compound **33**) [149]. This inhibitor show selectivity not only towards the CoREST complex but also to HDAC1 and HDAC2 at the monomeric protein level. In addition, low hematological side effects make Rodin-A a promising compound for neurologic disorders [149].

As mentioned in previous sections, some of the HDAC complexes (NuRD and CoREST) possess two different enzyme activities simultaneously. Dual-action inhibitors which contain two pharmacophores in a single molecule may target both activities of these enzymes [15]. A dual-action inhibitor, corin (Figure 9, Compound **34**), can effectively target the CoREST complex and show potential in treating many tumor cells [83]. 4SC-202 (domatinostat, Figure 9, Compound **35**) is another dual-action inhibitor designed for targeting the CoREST complex, currently under development in the treatment of colorectal cancer and hematological malignancies [15,150,151].

Additionally, disturbing the interface of adjacent subunits in HDAC complexes can be a good idea, as suggested in Schwabe’s review [15]. Waxman’s group found a decoy peptide can interfere with the binding interface of Sin3A/B and other partner proteins, which may explain its specific inhibitory effect on the Sin3 complex [154]. Latterly, they have screened out some compounds and peptides as the candidates for inhibiting the interaction of Sin3 protein with another partner protein, MAD [152,155].

Targeting subunits other than HDACs in these complexes also provide an idea. For example, resveratrol (Figure 9, Compound **36**) can decrease the expression of MTA1 in prostate cancer cells, which then reduces the amount of MTA1:HDAC1 complexes [146]. However, resveratrol is also an activator of SIRT1 with treatment potential in diverse diseases such as neurodegenerative diseases, cancers and cardiovascular diseases with distinct pharmacological mechanisms [156,157]. In this regard, resveratrol cannot be classified as a complex-specific inhibitor, but it provides a conception for drug design.

## 7. Conclusions and Perspectives

Class I HDACs have been wildly investigated, given their important roles in epigenetic regulation. The effectiveness of HDAC inhibitors has also been confirmed in many disease treatments such as cancers, neurological diseases and inflammations and infections [8]. This review highlights the structural studies in Class I HDACs and their complexes as well as pan-, isoform-selective and complex-selective inhibitor development. Due to the poor selectivity and undesirable side effects that occur in pan-inhibitors, the development of selective inhibitors has attracted the attention of investigators. For example, PCI-34051 specifically inhibits HDAC8 and induces apoptosis in T-cell lymphoma without increasing the acetylation of histone and a-tubulin, thus have less toxicity than the pan-inhibitors [55]. Currently, most developed selective inhibitors are towards HDAC8 owing to its specificity in structure and functions. HDAC complexes and/or isoform-selective inhibitors are growingly becoming the focus of HDACis development. However, two major challenges faced in the development of HDAC1/2/3-selective or complex-selective inhibitors are (1) high sequence similarity and homological identities in Class I HDACs; (2) the lack of structures of these intricate HDAC multiprotein complexes. In complex and adaptive biological systems, there is no single formula or framework that always works. However, targeted HDACis development from different perspectives is certainly more promising and allows us to learn the best approaches for drug development.

HDAC complexes and/or isoform-selective inhibitors are increasingly becoming the focus of HDACis development. In the future, more efforts should be put into resolving the structures of complete HDAC complexes, dynamic interactions between subunits and specific inhibition in overall complexes. An increasing number of structural methods have come into view, such as cryo-electron microscopy (cryo-EM) and structural MS approaches. For instance, as mentioned above, XL-MS has been used in analyzing Sin3 complex and modeling a structure of this complex at a global level [80]. In addition, XL-MS [158,159], hydrogen-deuterium exchange MS (HDX-MS) [160,161,162], native MS [163,164], native top–down MS [165,166] techniques have been more frequently used for the structural studies of macromolecular protein complexes [167]. The integration of different structural techniques is certainly enhancing the analysis of protein-inhibitors interaction and inhibitor designs. In summary, structure-based studies aid the disclosure of the mechanism of action of protein–drug complexes and conversely promote the development of HDAC inhibitors.

## Abbreviations

AML	Acute myeloid leukemia
AP-MS	Affinity purification mass spectrometry
CDK2AP1	Cyclin-dependent kinase 2-associated protein 1
CFDA	Chinese Food and Drug Administration
CHD	Chromodomain helicase DNA‑binding protein
CNS	Central nervous system
CoREST	Corepressor of RE1-silencing transcription
Cryo-EM	Cryo-electron microscopy
DLBCL	Diffuse large B-cell lymphoma
DNTTIP1	Deoxynucleotidyl-transferase terminal-interacting protein 1
EM	Electron microscopy
FDA	Food and Drug Administration
GPS	G-protein pathway suppressor
HDAC	Histone deacetylase
HDX-MS	Hydrogen-deuterium exchange MS
HL	Hodgkin lymphoma
Hsp90	heat shock protein 90
LSD	Lysine-specific demethylase
MBD	Methylated CpG-binding domain protein
MiDAC	Mitotic deacetylase
MIDEAS	Mitotic deacetylase-associated SANT domain
MTA	Metastasis tumor-associated protein
NCoR	Nuclear receptor corepressor
NMPA	National Medical Products Administration
NMR	Nuclear magnetic resonance
NuRD	Nucleosome remodeling and deacetylase
PTMs	Post-translational modifications
QM/MM	Quantum mechanical/molecular mechanical
RBBP	Retinoblastoma-binding protein
SAHA	Suberoylanilide hydroxamic acid
SAP30	Sin3-associated protein p30
SAXS	Small-angle X-ray scattering
Sin3	Switch intensive 3
SIRT	Sirtuin
SMRT	Silencing mediator of retinoic acid and thyroid hormone receptors
STAT3	Signal transducers and activators of transcription 3
SUD3	Suppressor of defective silencing 3
TBL1	Transducin beta-like 1
XL-MS	Chemical crosslinking mass spectrometry
ZBG	Zinc binding group
